# Cyclooxygenase-2 Inhibitor: A Potential Combination Strategy With Immunotherapy in Cancer

**DOI:** 10.3389/fonc.2021.637504

**Published:** 2021-02-26

**Authors:** Dan Pu, Liyuan Yin, Lin Huang, Changlong Qin, Yuwen Zhou, Qiang Wu, Yan Li, Qinghua Zhou, Lu Li

**Affiliations:** ^1^ Department of Lung Cancer Center, Lung Cancer Center, West China Hospital, Sichuan University, Chengdu, China; ^2^ Oncology Department, West China Hospital, Sichuan University, Chengdu, China

**Keywords:** cyclooxygenase-2, immunotherapy, cancer, combination therapy, celecoxib

## Abstract

The clinical application of immunotherapy is the milestone of cancer treatment. However, some patients have bad reaction. Cyclooxygenase-2 (COX-2) is frequently expressed in multiple cancer cells and is associated with poor prognosis. It is the key enzyme of prostaglandin E_2_ (PGE2) that has been proved to promote the development, proliferation and metastasis of tumor cells. Recent studies further find the PGE2 in tumor microenvironment (TME) actively triggers tumor immune evasion *via* many ways, leading to poor response of immunotherapy. COX-2 inhibitor is suggested to restrain the immunosuppression of PGE2 and may enhance or reverse the response of immune checkpoint inhibitors (ICIs). This review provides insight into the mechanism of COX-2/PGE2 signal in immunosuppressive TME and summarizes the clinical application and trials in cancer treatment.

## Introduction

Cancer is a global health problem. In recent years, immunotherapy has become a hot spot. ICIs, the most popular kind of immunotherapy, for variety of cancers have shown better efficacy than conventional chemotherapy. However, there are remaining questions needed to be resolved. The main two important questions were: Why the response to ICIs are different in different patients? How to increase the population benefiting from immunotherapy? Programmed death receptor-1 (PD-L1) expression, tumor infiltration lymph cells (TILs) and tumor mutation burden (TMB) are considered to be associated with ICIs efficacy (1). But even though the expression of PD-L1, the rate of TILs and the TMB are high in some patients, the efficacy of ICIs is still limited, which points out a non-PD-1/PD-L1 axis mediated immunosuppression (2). The leading theory explaining this phenomenon includes two aspects. One is the intrinsic factors, such as cancer-driven signaling pathways, MHC downregulation, microsatellite stability, etc. (3, 4). The another is extrinsic factors, known as TME. Cancer cells are closely related to extracellular matrix, stromal cells, and immune cells, which together constitute TME (5) and these components in TME impact the efficacy of ICIs, for example, the number of regulatory T cells (Tregs), myeloid suppressor cells (MDSCs), dendritic cells (DCs) and the activity of indoleamine 2,3-dioxygenase (IDO). TME is closely associated with inflammatory response and the inflammatory mediators in TME can be produced by stroma, TILs or cancer cells themselves. Prostaglandin E2 (PGE2) is one of the most important inflammatory factors in TME, which is related to the survival, growth, migration, invasion, angiogenesis and immune evasion of cancer cells (6). Cyclooxygenase (COX) is the rate-limiting step enzyme that produces PGE2. There are three isoforms: COX-1, COX-2, COX-3 (7). COX-1, constitutively expressed in a wide range of normal tissues, works as a housekeeping enzyme responsible for maintaining tissue homeostasis. COX-3 is a splice variant of COX-1, which is also called COX-1b or COX-1v. COX-2 barely presents in most normal cells, but can be highly induced by inflammation and cancer (8). Previous studies have shown that COX-2 is overexpressed in most cancers and is associated with poor prognosis (9, 10). With the rise of immunotherapy, more and more studies have shown that COX-2 mediates immunosuppression *via* multiple ways. This review summarizes the roles of COX-2 in the resistance of ICIs and proposes a position and opportunity for COX-2 inhibitors in combination with immunotherapy in cancers.

## The Activation of COX-2/PGE2 Pathway in Cancer

Gene encoding COX-2 is located on the chromosome 1q25.2‐q25.3 in human, known as *PTGS2*. COX-2 is a membrane-bound enzyme that plays a key role in synthesis of important biological hormones-prostaglandins (PGs), such as PGE2, PGF2α and thromboxane (11). COX-2 is usually negligible in normal cells except basal expressed in a few organs, such as stomach, kidney, central nervous and female reproduction. While it is frequently expressed in most types of cancers, including lung cancer (12), gastrointestinal cancer (13), breast cancer (14), head and neck carcinoma (15), hepatocellular carcinoma, and etc (16). COX-2 overexpression is linked to many properties of malignant cells including promoting carcinogenesis, increasing the rate of cancer recurrence, reducing survival and mediating resistance of tumor cells to treatment, through overproduction of PGs (17).

Oncogenic viruses, inflammatory cytokines can elevate the expression of COX-2. Tumor intrinsic factors also upregulate COX-2/PGE2 axis. Markosyan and his colleagues identified *PTGS2* was upregulated by EPHA2, a candidate tumor intrinsic driver of immunosuppression, through TGF-β pathway in pancreatic cancer (18). The reduction of RIPK3 in colorectal cancer cells and MDSCs elicited NF-κβ-transcribed COX-2, thus to exacerbate the immunosuppressive activity of MDSCs (19). In breast cancer, HDAC6 was frequently upregulated in the cancer-associated fibroblasts(CAFs) and increased the expression of COX-2/PGE2 by regulating STAT3 activation (20), leading to poor survival outcomes. In addition, the aberrant activation of EGF (21), KRAS (22), p38MAPK (23, 24) signals, which frequently present in cancers, also induce COX-2 expression, thus to mediate immunosuppression.

## The Mechanism of Immunosuppression Mediated by COX-2

Malignant cells can escape immune-surveillance by exhaustion of CD8+ T cells expressing programmed cell death protein 1 (PD-1) and cytotoxic T-lymphocyte-associated protein (CTLA-4). ICIs are a class of inhibitors that targeted immune checkpoint proteins marked on the surface of cancer cells, like CTLA-4 receptor and programmed cell death protein ligand 1 (PD-L1), so that to remove the inhibition of T cells by cancer cells. Previous evidences suggested that the impact of COX-2/PGE2 signal pathway in TME plays an important role in immunosuppression and further induces ICIs resistance. Long before the advent of immunotherapy in clinic, the role of COX-2/PGE2 in immunosuppression was deeply studied in laboratory.

### COX-2/PGE2 Signal Inhibits T Cell Infiltration

T cells infiltrated to tumors recognize and fight against antigen-targeted tumor cells. The non-T cell inflamed tumors are usually difficult to treat with ICIs (25). Markosyan showed that compared with wild-type, the onset of breast tumors in ErbB2 transgenic mice with mammary epithelial cell COX-2 deficiency (COX-2^MEK^KO) was delayed. COX-2^MEK^KO TME contained more CD4^+^ T helper cells and CD8^+^ cytotoxic T lymphocytes (CTL). The Th1 marker Tbet and Th2 marker GATA3 were overexpressed, while Retnla, the marker of M2 macrophage cell was lower expressed in COX-2^MEK^KO tumor than the wild-type, suggesting an enhanced immune-surveillance (26).

Analysis of samples from human and mouse cancer cells with low or high TIL density again confirmed the association between COX-2 expression and T cell exclusion. In pancreatic cancer (18), the intrinsic TGF-β signaling of tumor cells drives the upregulation of EPHA2 on cell surface, which promotes the overexpression of *PTGS2*. In vivo study, researchers inoculated control and *Ptgs2*-knockdown (*Ptgs2*-KD) cells into immunocompetent mice, with or without CD4^+^ and CD8^+^ T cell depletion, resulting in a higher rate of tumor formation in control cells. T cell depletion abolished the tumor growth suppression afforded by *Ptgs2* KD or celecoxib, which indicates that the tumor suppression of *PTGS*2 is T cell dependent. In *PTGS2* overexpression tumors, the proportion of CD4^+^ and CD8^+^ T cells and the percentage of activated CD8^+^ T cells were significantly reduced. In addition, *PTGS2* overexpression increased the proportion of infiltrated myeloid cells, especially myeloid-derived suppressor cells (MDSCs), with a decrease in the dendritic cells (DCs) population.

A series of human cancer cells constitutively express indoleamine 2,3-dioxygenase 1 (IDO1) that degrades tryptophan and produces equimolar amounts of kynurenine, also mediates immunosuppression. Marc Hennequart’s study indicated that COX-2 expression drives the constitutive expression of IDO1. In human tumor cell lines, constitutive IDO1 expression depends on COX-2 and PGE2 *via* EP receptor through PKC and PI3K pathways. Celecoxib treatment decreased IDO1 expression and increased CD3^+^ and CD8^+^ cells infiltration in ovarian SKOV3 tumors (27).

### COX-2/PGE2 Signal in NK-DC Crosstalk

Nature kill (NK) cell as a part of innate immunity plays an important role in tumor immune surveillance. NK cells not only directly recognize and kill tumor cells, but also release cytokines that promote CTL activation and proliferation. Park. A and his colleagues showed thyroid cancer-derived PGE2 represses NK maturation and the expression of NK receptors, such as NK44, NK30, TRAIL and NKG2D (28). Inhibition of COX-2/PGE2 signal pathway can recover the activation of NK cells in tumor-bearing mice (29). Besides, Böttcher revealed the intercommunication between NK cell and dendritic cells (DCs) (30). NK cells recruit conventional type 1 DCs (cDC1) by release of CCL5 and XCL1. Tumor-derived PGE2 impairs NK cell viability and chemokine production, then decreases the recruitment of cDC1s to TME. DCs, especially Batf3^+^ CD103^+^ cDC1, are essential in presenting tumor antigen and secreting cytokines, such as CXCL9, CXCL10, that regulate T cell function (31). CD103^+^ DCs were selectively absent in tumor expressing COX-2. In an obesity-associated hepatocellular carcinoma mice model, the daily systemic therapy of PGE2 receptor inhibitor for 3 weeks showed significant induction of cDC1 (CD103^+^ DC) frequency (32).

### COX-2/PGE2 Signal Induces MDSCs

MDSCs can inhibit CTL activation by overexpression of argininase 1 (ARG-1), inducing nitric oxide synthase (iNOS or NOS2) and reactivating oxygen species (ROS), thus inducing immune escape. COX-2/PGE2 signal pathway is associated with the accumulation of MDSCs. In colorectal cancer, the reduction of PIRK3 elicited NF-κβ transcribed COX-2 expression and boosted the synthesis of PGE2. Inhibition of COX-2 or PGE2 receptors reversed the immunosuppressive activity of MDSCs and dampened tumorigenesis (19). Porta et al. (33) also demonstrated tumor-derived PGE2 mediated induction of nuclear p50 NF-κB epigenetically reprograms the response of monocytic cells to IFN-γ toward an immunosuppressive phenotype, thus retrieving the anticancer properties of IFN-γ. Inhibition of the PGE2 axis can prevents MDSC suppressive functions and restores the efficacy of anticancer immunotherapy.

Multiple tumor cell lines, like Braf V600E melanoma, 4T1 breast cancer, CT26 colorectal cancer, *Nras* G12D-drive mouse melanoma, methylcholine-induced fibrosarcoma (34, 35) aberrant expressed COX-2/PGE2. The conditional medium of Braf V600E melanoma cells regulated the function of myeloid cells by expressing COX-2 and PGE2. COX-2 deficiency resulted in low expression of immunosuppression factors like IL-6, IL-10, and CXCL1, while the mRNA of anti-tumor immune mediators are significantly increased, such as IFN-γ, T-bet, CXCL10, and IL-12 (36).

MDSCs can also negatively regulate NK function. MDSCs from patients with advanced melanoma inhibited the activity of co-cultured NK cells. PGE2 binding to EP2 and EP4 receptors on MDSC activates p38MAPK/ERK pathway, leading to TGF secretion and thereby inhibiting NK cells (37).

### COX-2/PGE2 Signal Induced M2

Macrophages are the most plastic cells in the hematopoietic system, which are found in all tissues, and they also have strong functional diversity. There are at least two subtypes of macrophages, namely M1 and M2. M1 macrophages are involved in the pro-inflammatory response and play a central role in the host’s defense against bacterial and viral infections. M2 macrophages are associated with resolution of inflammatory response, parasite infection, tissue remodeling, fibrosis, and tumor disease development. Previous studies had pointed out an important role of PGE2 in the polarization of macrophage to M2, leading to an immunosuppression TME. In vitro experiments, a human peripheral blood mononuclear cell primary culture in the presence of GM-CSF plus IL-4 promoted differentiation to DCs. An addition of PGE_2_ in this culture suppressed the formation of DCs and skewed the differentiation into the M2-like macrophage (38, 39). PGE2 also induces the differentiation from MDSC to M2 macrophage. The cross-talk between miR-21 and PGE_2_ may be a determining factor in macrophage polarization. PGE2 and its downstream effectors PKA and Epac inhibited mRNA-21 and enhance the expression of M2 gene (40).

**Figure 1:**
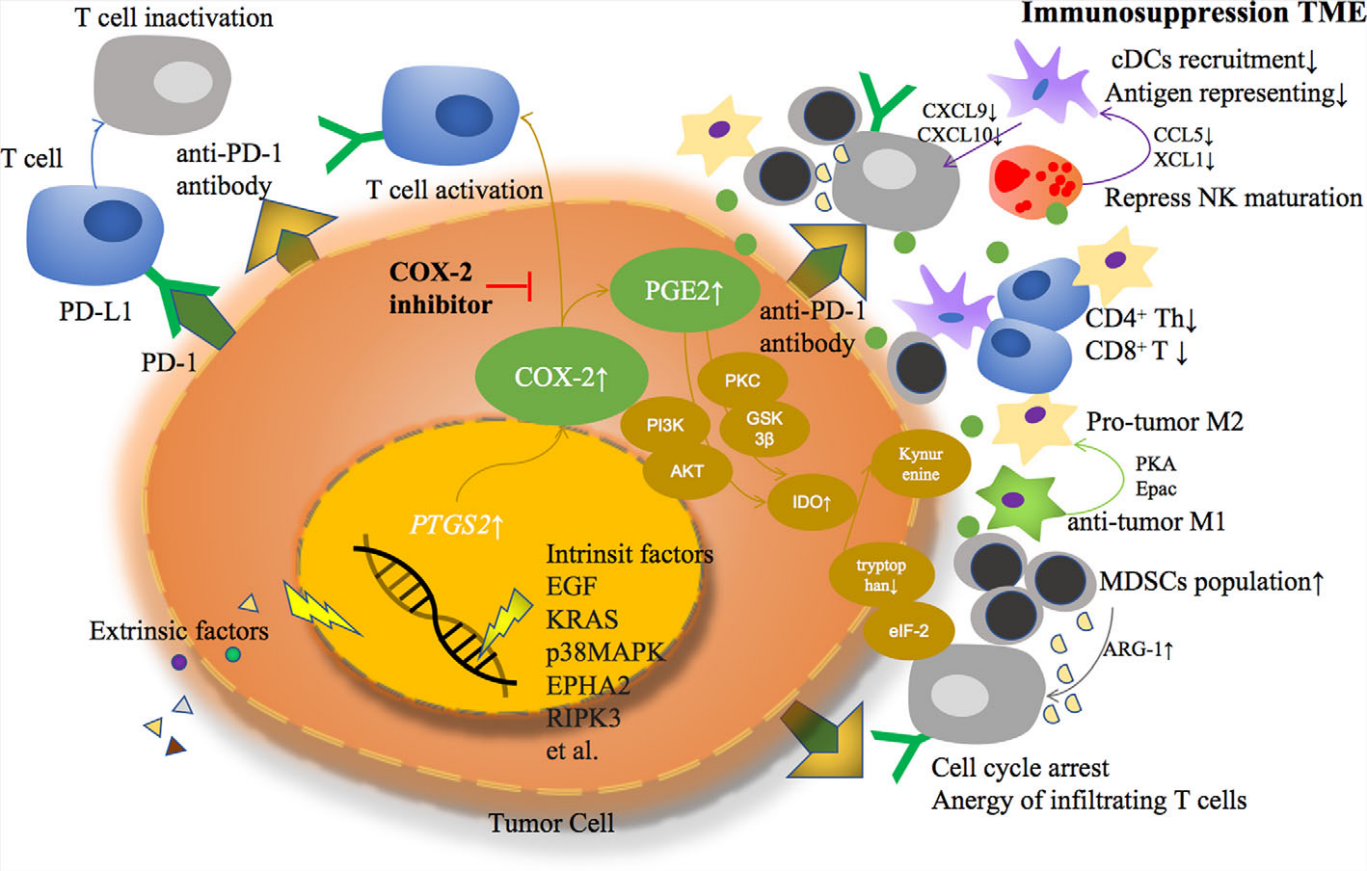
Extrinsic or intrinsic factors lead to COX-2 overexpression and PGE2 over production in tumor cells, formatting an immunosuppressive tumor microenvironment through multiple ways.

Based on previous studies, COX-2 derived PGE2 helps TME transformed from an anti-tumor response to an immunosuppressive response in a variety of ways, becoming an accomplice of cancer cell immune escape (**Figure 1**).

## Clinical Application of COX-2 Inhibitors in Cancer Treatment

The current focus of immunotherapy is to improve the therapeutic response of ICIs by simultaneously stimulating immune function and targeting immunoregulatory factors in TME. A variety of combinations are in clinical trials, such as ICIs combined with GM-CSF, targeted drugs, oncolytic virus, chemotherapy, radiotherapy, IDO inhibitor, etc. (41, 42).

Researchers have long noticed the important role of COX-2 in the occurrence and development of cancer. Once upon a time, the clinical trials of COX-2 inhibitor combination therapy in cancers were popular. However, no satisfactory results were obtained in such combinations. A phase II clinical trial of COX-2 inhibitor combined with erlotinib, one of the epidermal growth factor receptor tyrosine kinase inhibitor (EGFR TKI), seemed to have an increase trend in time to progression (TTP) and overall survival (OS) in non-small cell lung cancer (NSCLC) (43). While compared with conventional chemotherapy regiments, combined with COX-2 inhibitor did not achieve survival improvement (44, 45). The conclusion was controversial. Csiki et al. (46) performed a stratified analysis of the decreased levels of urine PGE-M (the main urinary metabolite of PGE2) after using celecoxib, and showed that patients with a large decline rate obtained a longer survival (14.8 months, 6.3 months, 5.0 months, respectively). Are COX-2 inhibitors really useful in cancer treatment? COX-2 expression level, metabolites or COX-2-dependent inflammatory mediators may be useful biomarkers for predicting prognosis and outcomes of combination therapy. Based on the mechanism of COX-2 and PGE2 in TME described above, the combination of COX-2 inhibitor and ICI is a potential choice. This idea has been further verified in animal experiments (36). The combination of COX inhibitors (including aspirin and celecoxib) with anti-PD-1 antibodies can promote tumor regression more than the single use of anti-PD-1 antibodies. What’s more, it has been known that ICI combined with chemotherapy would increase disease control, because of the antigen release induced by chemotherapy. So the benefit of COX-2 inhibitor might be based on the immune activation combined with ICI and chemotherapy plays a supporting role in it, which need to be further study in clinical practice.

In order to further find patients who may benefit from this combination, the relationship between COX-2 and cancers in previous studies were also reviewed. An analysis of 170 cases of surgically resected lung adenocarcinoma showed that high COX-2 expression accounted for 46%, and the number of CD8^+^ T lymphocytes in tumors with high COX-2 expression was significantly less than that in the low expression group, while the Treg count was in the opposite (47). Shimizu et al. reported correlations between COX-2 and immune checkpoint proteins. Double fluorescence staining showed co-localization of PD-L1 and COX-2 expression in resected lung cancer specimens (48). Besides, Kim’s study analyzed the relationship between PD-L1 RNA and COX-2 expression in 60 human melanoma cell lines in CCLE database and also showed a significant correlation (r=0.312, P=0.014). But *in vitro*, COX-2 inhibitor, celecoxib did not affect the expression of PD-L1 induced by IFN-γ in melanoma cell lines, A375, SB2 and LOX-IMVI (49). So, COX-2 might be an intrinsic characteristic of certain cancer cells, and mediates immunosuppression *via* not only PD-1/PD-L1 axis.

In terms of the safety of ICI combined with COX-2 inhibitor, several aspects should be taken into consideration. First of all, ICIs are a kind of monoclonal antibody. Their metabolic pathways are similar to that of endogenous IgG, not go through the cytochrome P450 enzyme metabolic pathway. While celecoxib is mainly metabolized by CYP2C9 (50), so there might be no drug interaction between them theoretically. Secondly, we take Celecoxib, the most representative selective COX-2 inhibitor, as an example, to illustrate the side-effects of COX-2 inhibitor. The most common side-effects of Celecoxib at a dose of 400–800 mg/day for 3 years are diarrhea (10.5% in Celecoxib group vs. 7.0% in placebo), Gastroesophageal reflux (4.7% in Celecoxib group vs. 3.1% in placebo), nausea (6.8% in Celecoxib group vs. 5.3% in placebo), vomit (3.2% in Celecoxib group vs. 2.1% in placebo), dyspnea (2.8% in Celecoxib group vs. 1.6% in placebo), hypertension (12.5% in Celecoxib group vs. 9.8% in placebo). The occurrence of heart abnormalities and thrombotic event were between 0.1% and 1%. The previous trials of celecoxib combined chemotherapy regimens were well tolerated and did not show an increase in serious adverse events (45, 51). Especially when compared with the placebo group, there was no increase in cardiovascular events in celecoxib group (45). Finally, it is very likely that the main risks of the combination therapy of COX-2 inhibitor and ICI come from their own separately. According to the dose of celecoxib used in CLASS study, the incidence of complicated and symptomatic ulcers was only 0.78% and the incidence of severe cardiovascular thromboembolic events was only 1.2% continuously taking 400 mg twice daily for 9 months (52). And Csiki et al. (46) have also shown that at this dose intensity, urine PGE-M is significantly reduced, indicating that COX-2 and its derived PGE2 are significantly restrained.

Some clinical trials about COX-2 inhibitor combination therapies are ongoing (**Table 1**). In colorectal cancer, trials to evaluate PD-1 inhibitors with celecoxib as neoadjuvant therapy are recruiting (NCT03026140, NCT03926338). In breast cancer, NCT04188119 and NCT04348747 are registered. There are also some other combined treatments in progress. For example, RACIN (NCT03728179) is designed to explore the combination of PD-1 inhibitor with or without CTLA-4 inhibitor, aspirin (non-selective COX inhibitor) or celecoxib (selective COX-2 inhibitor) and low-dose radiotherapy in TIL negative solid tumors, which might answer the immunomodulation effect of aspirin or celecoxib. Selective COX-2 inhibitor induces less gastrointestinal reaction, one of the most common side-effects of COX inhibitors, like gastric ulcer. What’s more, a large clinical study in 2017 showed that compared with the COX-2 selective inhibitor Celecoxib, the non-selective COX inhibitors ibuprofen and naproxen significantly increased the systolic blood pressure, and the occurrence of new hypertension was higher (53). In fact, taking Celecoxib or non-selective ones (such as ibuprofen) for up to three years have shown that the risk of cardiovascular events increases. So, theoretically, selective or non-selective COX inhibitor, as long as drugs targeting COX2/PGE2 signal pathway, could be further studied in clinical practices, but selective one at least reduces gastrointestinal reaction. Thus the side effect of COX-2 inhibitor should be taken into consideration carefully and exclude patients that have contraindications. The appropriate dose of these drugs is uncertain by now. Some scientists considered that whether PGE2 receptor inhibitor, instead of COX-2 inhibitor, could also combined with immunotherapy. The COX-2 product PGE2 binds to four G-protein-coupled EP receptors designated EP1-EP4. Recent drugs7nbsp;only designed to block EP4. EP4 is commonly upregulated in cancers, while MDSCs are induced by PGE2 acting on myeloid-expressed EP2 and EP4 (54). So, the effect of EP4 inhibitor combined with ICI remains to be seen. Anyway, the COX-2/PGE2 signal is a promising target in combination with immunotherapy.

**Table 1:** The clinical trials that study the COX inhibitor and immunotherapy registered in clinicaltrial.gov.

Clinical Trial	Cancer	Therapy	Phase	Status
NCT03026140	Colon carcinoma	Ipilimumab, Nivolumab; Ipilimumab, Nivolumab, Celecoxib	II	Recruiting
NCT04188119	Breast cancer	Avelumab, PPI; Avelumab, Aspirin, PPI	II	Not yet recruiting
NCT03926338	Colorectal cancer	PD-1 inhibitor, Celecoxib; PD-1 inhibitor	I, II	Recruiting
NCT04348747	Breast cancer	DC vaccine, pembrolizumab, celecoxib, IFNα-2b,rintatolimod	II	Not yet recruiting
NCT03728179	TIL-negative solid tumors	Low dose irradiation+Nivolumab+Ipilimumab or Cyclophosphamide + Aspirin/Celecoxib	I	Recruiting
NCT03245489	Squamous Cell Carcinoma of the Head and Neck	Pembrolizumab, Clopidogrel, Aspirin; Pembrolizumab	I	Recruiting

TIL, Tumor Infiltrating Lymphocytes; PPI, Proton Pump Inhibitor.

## Conclusion

Given that COX-2/PGE2 axis promotes immunosuppression, it is conceivable that COX inhibitors have a role in anti-tumor therapy. Unfortunately, former clinical attempt of combination COX inhibitor with chemotherapy or targeted therapy failed. But COX inhibitor might enhance or expand the response of immunotherapy in consideration of its mechanism. Several clinical trials are ongoing. They will provide us a new thought of therapeutic approach in cancer immunotherapy.

## Author Contributions

DP wrote the manuscript. LY, LH, and CQ searched the references. YZ, QW, and YL contributed to the scientific discussion. QZ modified the manuscript. LL supported the research. All authors contributed to the article and approved the submitted version.

## Funding

2020HXFH046, 1·3·5 Project for Disciplines of Excellence-Clinical Research Incubation Project, West China Hospital, Sichuan University.

## Conflict of Interest

The authors declare that the research was conducted in the absence of any commercial or financial relationships that could be construed as a potential conflict of interest.
